# Hyponatremia correction is associated with increased brain‐derived neurotrophic factor levels: A pilot secondary analysis of a randomized, double‐blind, placebo‐controlled, crossover trial

**DOI:** 10.1111/jne.70238

**Published:** 2026-07-28

**Authors:** Eszter Kustos‐Tóth, Julia Beck, Lucia Seeger, Sophie Monnerat, Cemile Bathelt, Julie Refardt, Mirjam Christ‐Crain

**Affiliations:** ^1^ Department of Endocrinology University Hospital Basel Basel Switzerland; ^2^ Department of Internal Medicine University Hospital Basel Basel Switzerland; ^3^ Department of Clinical Research University Hospital Basel, University of Basel Basel Switzerland

**Keywords:** brain‐derived neurotrophic factor (BDNF), cognition, hyponatremia, syndrome of inappropriate antidiuresis (SIAD)

## Abstract

Chronic hyponatremia is associated with cognitive deficits, yet the underlying mechanisms remain elusive. A cerebral growth factor which is crucial for cognition and memory is brain‐derived neurotrophic factor (BDNF), but its role in hyponatremia has never been investigated. We here examined the influence of hyponatremia correction on serum BDNF levels. This study represents a secondary post hoc analysis of a prospective randomized, double‐blind, crossover, placebo‐controlled trial. Nine patients with syndrome of inappropriate antidiuresis (SIAD) completed 4 weeks treatments with empagliflozin 25 mg/day versus placebo. Serum sodium, BDNF levels, and Montreal Cognitive Assessment (MoCA) were assessed at baseline and after treatment cycles. Median baseline sodium was 131 mmol/L [130–133], which increased to 134 mmol/L ([131–136], *p* = .04) after empagliflozin treatment and remained stable after placebo phase (130 mmol/L [126–132], *p* = .9). Sodium increase was significantly associated with BDNF increase, also after adjusting for treatment arm, sex, age, baseline sodium, and antidepressant treatment (per 1 mmol/L sodium increase: estimate = 0.70 ng/mL, *p* = .048). This association was stronger upon empagliflozin treatment as shown by the interaction analysis (*p* = .03). In a subgroup of patients reaching normonatremia after empagliflozin treatment, BDNF increase was slightly higher compared to patients with persistent hyponatremia. MoCA scores did not differ between empagliflozin and placebo phase. Sodium increase was significantly associated with an increase in neurocognitive biomarker BDNF. Further research is needed to clarify BDNF's role in cognitive impairment in hyponatremia and its response to different treatment approaches.

Trial registration: ClinicalTrials.gov (NCT03202667).

## 1 BACKGROUND

The brain‐derived neurotrophic factor (BDNF), identified in the 1980s, is a key growth factor of the central nervous system (CNS), synthesized by neurons and microglial cells.^1^ Data indicate that BDNF plays an important role in neuroprotection and neuroplasticity^2^ via receptor‐mediated intracellular signaling pathways.^3^ Peripheral BDNF levels have been shown to correlate with central BDNF levels^4^ and cognitive performance^5^, ^6^, ^7^ partially due to BDNF's bidirectional permeability across the blood–brain barrier.^8^ Reduced BDNF levels are associated with neurodegeneration and the pathogenesis of major disorders, including Alzheimer's disease, depression, schizophrenia, bipolar disorder, and anxiety.^9^ On the other hand, BDNF plays a major role in learning and memory^10^, ^11^ with increased expression following antidepressant treatment^12^, ^13^ and physical activity.^14^

Hyponatremia is the most common electrolyte disturbance seen in clinical care,^15^ with the syndrome of inappropriate antidiuresis (SIAD) being the leading cause of euvolemic hyponatremia.^16^ Chronic hyponatremia is associated with increased morbidity, gait disturbances, osteoporosis,^17^ and cognitive impairment.^18^, ^19^ There is growing evidence that even patients with only mild hyponatremia (sodium < 135 mmol/L) show impaired cognitive performances.^17^, ^20^ Renneboog showed that the response latency of eight attention tests was significantly prolonged in patients with hyponatremia compared with normal serum sodium levels.^21^ The most profound changes have been observed in general attention decline and multisensory integration as shown by the “Phasic Alert” test, “Intermodal Comparison,” and the “Go/No Go” test. Importantly, studies have reported a partial reversibility of these cognitive impairments following correction of hyponatremia, underlining the causal relationship between hyponatremia and cognitive impairment.^20^, ^21^, ^22^

We have previously shown the efficacy of sodium‐glucose cotransporter‐2 (SGLT2) inhibitor empagliflozin in raising sodium levels in outpatients with chronic SIAD‐induced hyponatremia. In our randomized, double‐blind, placebo‐controlled cross‐over trial,^23^ empagliflozin treatment did not only lead to a sodium increase, but exploratory analyses also pointed towards an improved neurocognitive function in the Montreal Cognitive Assessment (MoCA) score after empagliflozin treatment.

Given the established associations between neurocognition and BDNF levels, and between neurocognition and hyponatremia correction, this secondary analysis aimed to investigate how hyponatremia correction affects serum BDNF levels.

## 2 METHODS

### 2.1 Study design

This is a secondary post hoc analysis of a prospective randomized, double‐blind, crossover, placebo‐controlled trial performed at the University Hospital Basel, Switzerland from 12/2017 to 08/2021. The primary objective of this secondary analysis was to investigate how hyponatremia correction affects serum BDNF levels.

The previously published, main study compared a 4‐week treatment with empagliflozin 25 mg/day vs. placebo in 14 outpatients with chronic SIAD‐induced hyponatremia. The complete information on the study's rationale, design, and statistical analysis has been published previously.^23^ In brief, participants were randomly assigned to undergo the first treatment period in the empagliflozin or, with equal chance, the placebo group. Treatment involved one tablet per day (empagliflozin 25 mg or placebo, respectively) for 28 days. All patients received a limitation of daily fluid intake of 1.5 L/day. A wash‐out period of at least 2 weeks between the treatment cycles was ensured. At baseline and after both treatment cycles, patients underwent clinical assessments including blood sampling and neurocognitive testing (MoCA). The MoCA is a 30‐point test (>26 defined as normal) used to assess the cognitive domains visuospatial, executive function, naming, memory, attention, language, abstraction, delayed recall, and orientation (to time and place).^24^

The trial was registered at ClinicalTrials.gov (NCT03202667). The local ethics committee (EKNZ 2017‐00701) as well as the national agency for the authorization and supervision of therapeutic products (swissmedics 2017DR2127) approved the study protocol and study medication.

### 2.2 Participants

**FIGURE 1:**
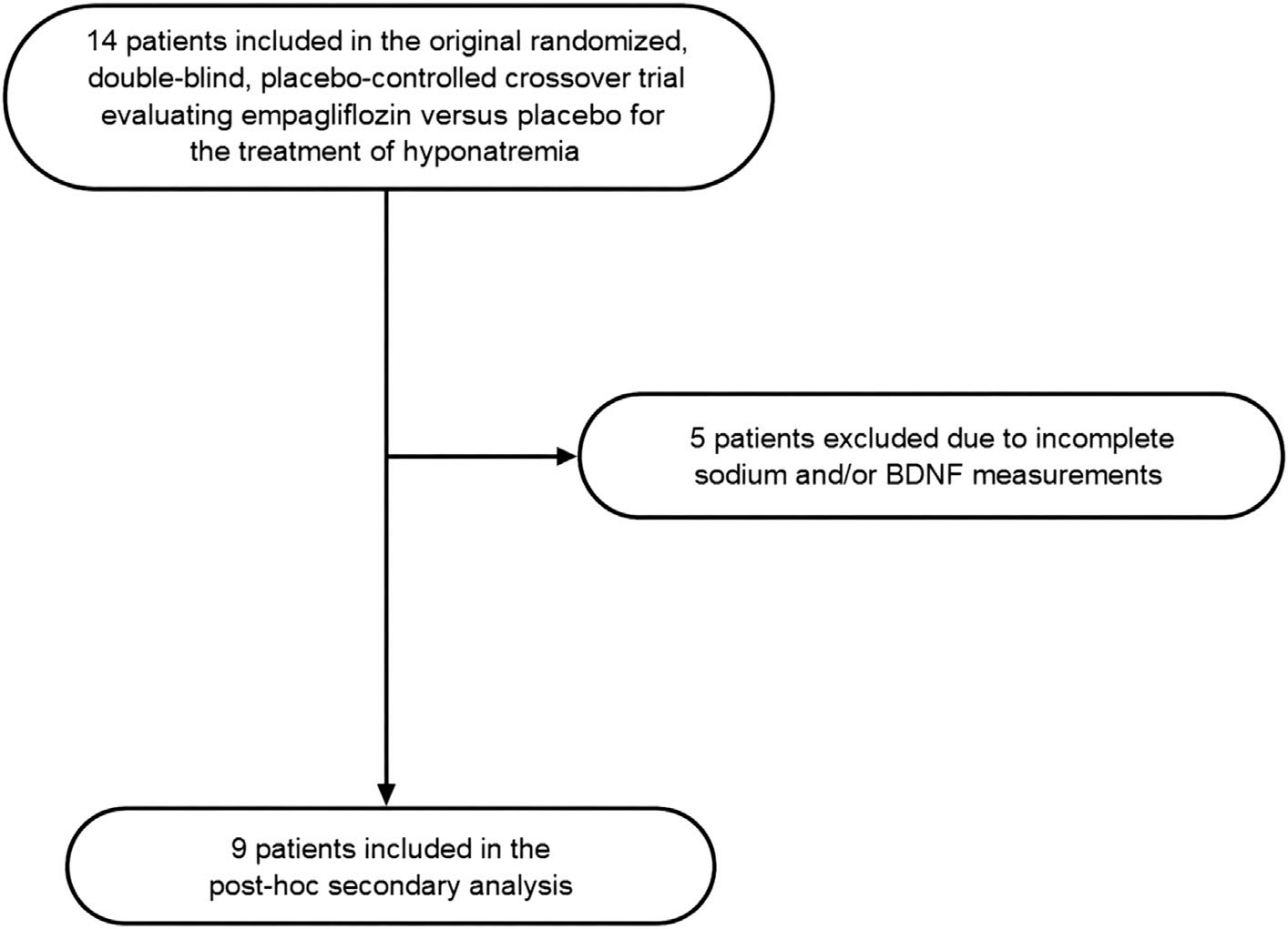
Flow diagram of patient selection for the post hoc secondary analysis.

Eligible patients were 18 years of age or older and had a chronic SIAD‐induced hyponatremia <135 mmol/L defined as: euvolemia according to clinical assessment, serum osmolality <275 mmol/kg, urine osmolality >100 mmol/kg, urine sodium >30 mmol/L, exclusion of hypothyroidism and hypocortisolism. Patients with acute or transient hyponatremia, severe symptomatic hyponatremia in need of hospital treatment, diabetes mellitus type 1, renal insufficiency (glomerular filtration rate (GFR) <45 mL/min/1.73 m^2^), heart failure, known liver cirrhosis or acute hepatic impairment (ALAT/ASAT > 3× upper limit), pregnancy or breastfeeding or patients under treatment with SGLT2 inhibitors, lithium chloride, urea or glitazone were excluded. For this secondary analysis, only patients with complete sodium and BDNF data available at each timepoint were included, resulting in an analytical cohort of 9 out of the 14 patients enrolled in the original trial (Figure 1). Subgroup analyses included sex and age groups, as well as differences between patients reaching normonatremia (≥135 mmol/L) and those remaining hyponatremic after treatment (<135 mmol/L).

### 2.3 Laboratory

Serum samples drawn in the fasting state were collected in a secure container (Sarstedt Monovette®), centrifuged, and stored at –80°C in a thermo‐controlled ultra‐deep freezer until the batch measurements were performed. BDNF concentrations from these previously frozen samples were analyzed at baseline and at the end of each treatment cycle. Samples were diluted 1:16. DPP4‐Inhibitor was added as advised. Quantitative enzyme‐linked immunosorbent assay kits from Meso Scale Diagnostics were used for BDNF measurements. The intra‐assay and inter‐assay CVs of this kit are reported as 3.0% and 10.1%, respectively.

### 2.4 Statistical analysis

Baseline characteristics were summarized using descriptive statistics. Discrete variables were expressed as frequencies (percentage (%) and number of patients (*n*)). Continuous variables were presented as median and interquartile range (IQR, 25th–75th percentiles). Differences between groups were tested using Wilcoxon Signed‐Rank test for paired data and Wilcoxon Rank‐Sum test for independent groups. Linear mixed‐effects models were used for regression models of repeated measurements. To account for the crossover design, each patient was included as a random effect (random intercept). An interaction model with an interaction term between sodium change and treatment arm was applied to assess whether the association between changes in sodium and changes in BDNF was modified by treatment arm (lmer function, R package *lme4*). To further examine the effect of sodium correction under empagliflozin treatment, an exploratory subgroup analysis was performed, assessing differences between participants reaching normonatremia (sodium ≥135 mmol/L) compared to participants remaining hyponatremic after the treatment period.

All data were presented visually by boxplots and scatterplots. Hypothesis testing was two‐sided, and *p*‐values <.05 were considered statistically significant. All analyses were performed in R version R 4.4.2 or higher (R Core Team, 2022. R: a language and environment for statistical computing, http://www.r-project.org/index.html).

## 3 RESULTS

A total of nine patients were included in the analysis; two‐thirds of the participants were female, median age was 74 years [63–76]. Main comorbidities were hypertension (89%), cerebrovascular disease, pulmonary disease, and epilepsy (22%). Seventy‐eight percent of patients were taking antihypertensive medication, 22% antidepressant treatment (Table 1).

**TABLE 1:** Baseline characteristics.

Characteristics	Participants (*n* = 9)
Age, years	74 [63; 76]
BMI, kg/m^2^	23.2 [20.3; 27.8]
Female sex, *n* (%)	6 (67%)
Duration of hyponatremia, months	47 [18; 78]
Sodium (mmol/L)	131 [130; 133]
Causes of SIAD, *n* (%)	
Central nervous system disorders	1 (11%)
Drug‐induced	3 (33%)
Pulmonary disease	2 (22%)
Idiopathic	3 (33%)
Comorbidities, *n* (%)	
Arterial hypertension	8 (89%)
Cerebrovascular disorder	2 (22%)
Pulmonary disease	2 (22%)
Epilepsy	2 (22%)
Diabetes mellitus type 2	1 (11%)
Multiple sclerosis	1 (11%)
Depression	1 (11%)
Drugs, *n* (%)	
Antihypertensive	7 (78%)
Statins	4 (44%)
Antiepileptic/multiple sclerosis treatment	3 (33%)
Antidepressant (Mirtazapine)	2 (22%)
Hormonal replacement	1 (11%)
Oral antidiabetics	1 (11%)

*Note*: Total cohort of *n* = 9 participants. Values are shown as median [IQR] for continuous variables or *n* (%) for categorical variables.

Abbreviations: BMI, body mass index; SIAD, syndrome of inappropriate antidiuresis.

**FIGURE 2:**
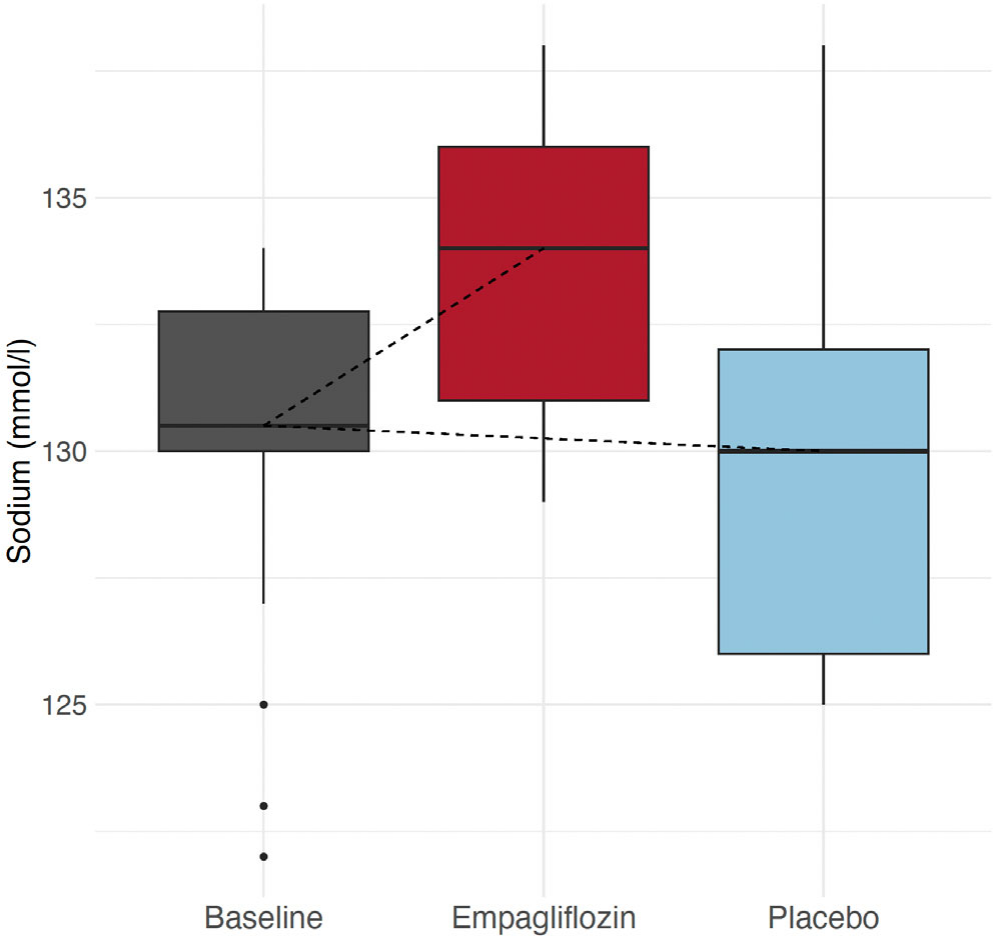
Sodium levels. Median sodium levels at baseline (131 [130–133]), after 4 weeks empagliflozin phase (134 mmol/L [131–136], *p* = .04) and after 4 weeks placebo phase (130 mmol/L [126–132], *p* = .9).

Sodium levels at baseline and after 4 weeks treatment phases are shown in Figure 2. Median serum sodium at baseline was 131 [130–133], which increased by +3 mmol/L after the empagliflozin phase to 134 mmol/L [131–136], *p* = .04. Sodium levels remained stable after placebo phase (130 mmol/L [126–132], *p* = .9).

**FIGURE 3:**
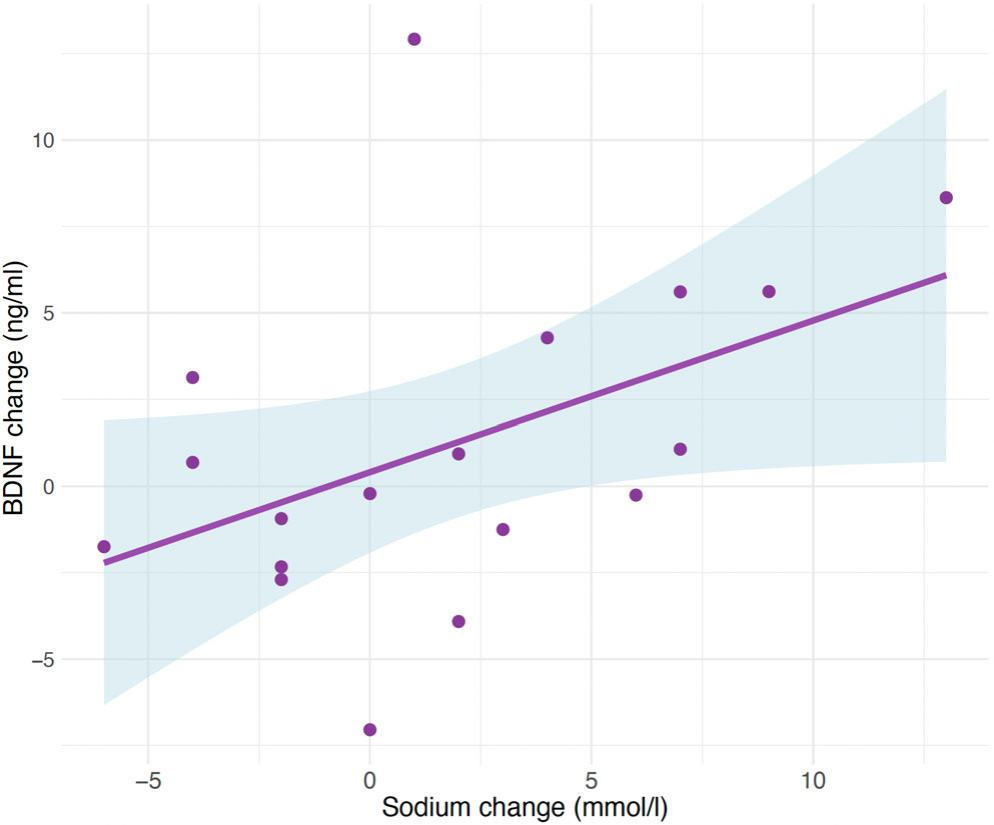
Relationship between sodium change and BDNF change. The sodium change was significantly associated with BDNF changes (estimate = 0.70 ng/mL per 1 mmol/L sodium increase, *p* = .048), after adjusting for treatment arm, sex, age, baseline sodium, antidepressant therapy and the cross‐over design. Statistics: linear mixed‐effects model, *n* = 18 observations (statistics: Table S1).

In the total cohort, an increase in sodium was significantly associated with an increase in BDNF levels (per 1 mmol/L sodium increase: estimate = 0.44 ng/mL, *p* = .05) (Figure 3). This association remained significant after adjusting for the treatment arm, sex, age, baseline sodium, and antidepressant therapy (per 1 mmol/L sodium increase: estimate = 0.70 ng/mL, *p* = .048).

**FIGURE 4:**
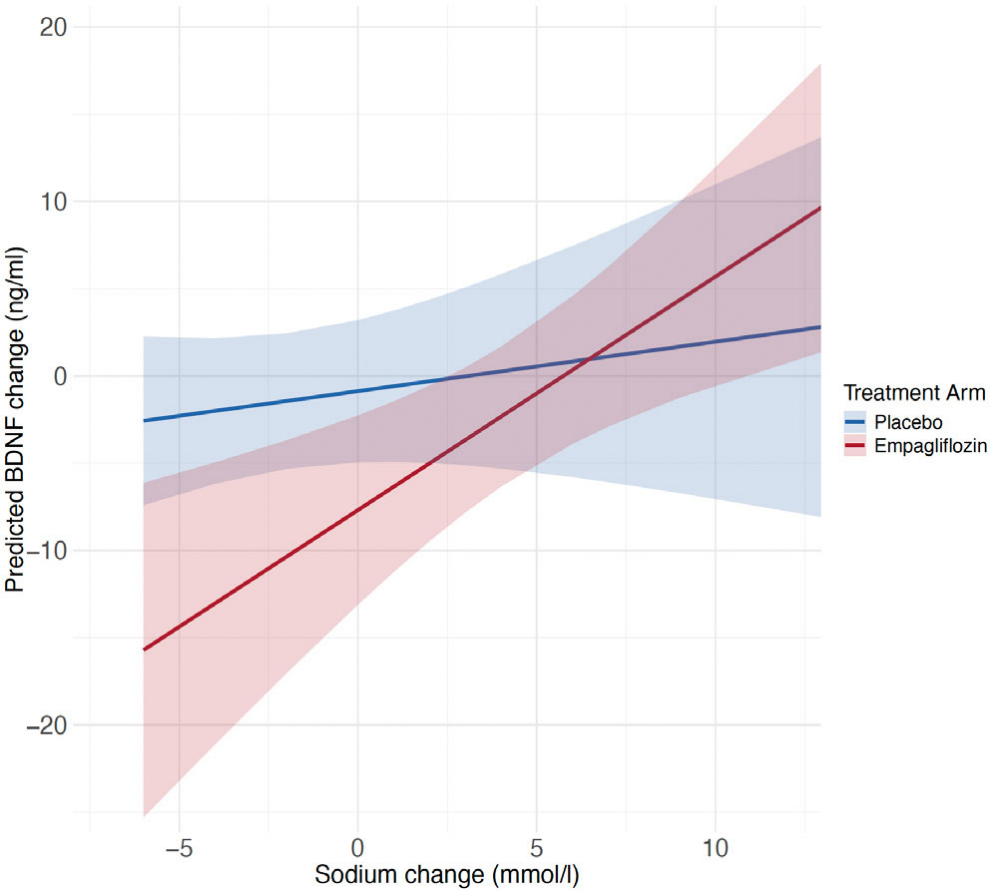
Interaction plot. Interaction analysis showing a significant interaction between the treatment arm and sodium change (*p* = .03), visualized by a stronger slope in empagliflozin than placebo in the interaction plot (empagliflozin: +1.34 ng/mL BDNF increase per mmol/L sodium increase, placebo: +0.28 ng/mL). Statistics: linear mixed‐effects model with an interaction term between sodium change and treatment arm. *n* = 18 observations.

There was a significant interaction between the treatment arm and sodium change (*p* = .03). An increase of 1 mmol/L in sodium was associated with a 1.34 ng/mL increase in BDNF under empagliflozin, whereas under placebo the same sodium increase was associated with a smaller increase of only 0.28 ng/mL. This suggests that empagliflozin amplifies the BDNF response to sodium changes, visualized by a stronger slope under empagliflozin treatment compared to placebo in the interaction plot (Figure 4).

**FIGURE 5:**
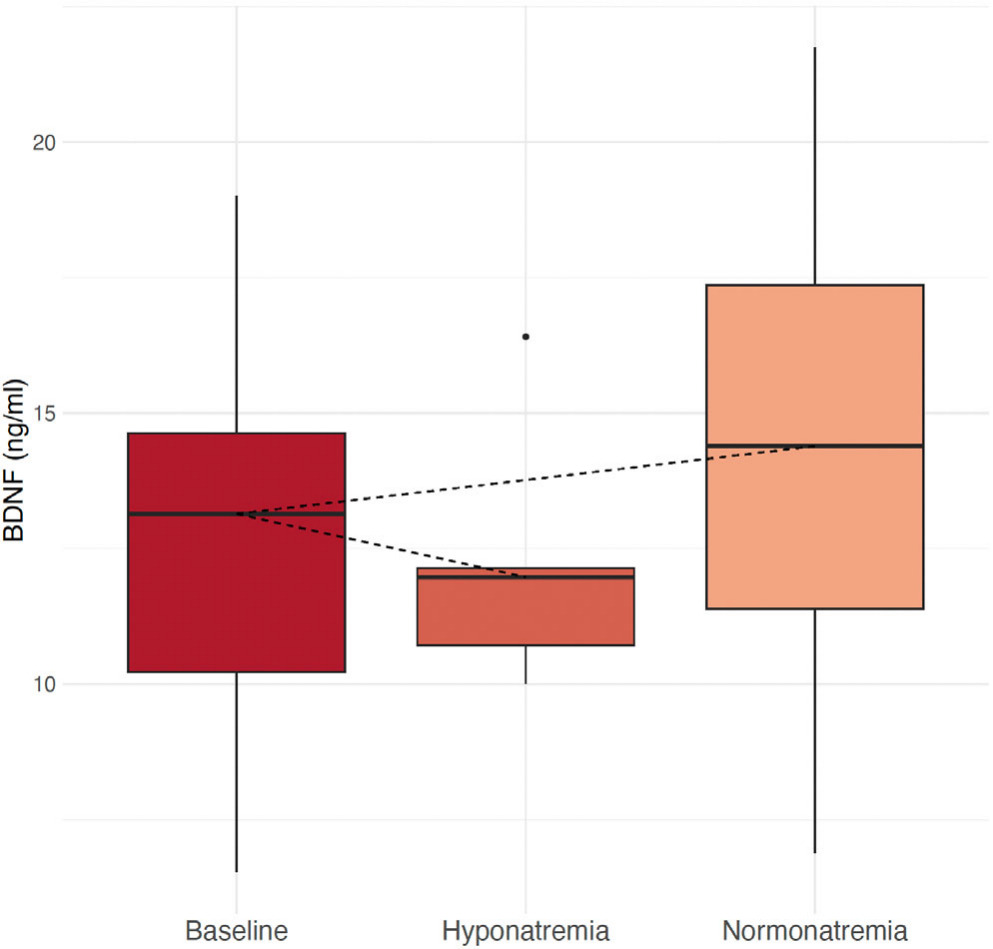
Subgroups reaching normonatremia versus remaining hyponatremic. Subgroup analysis of patients reaching normonatremia (*n* = 4, sodium ≥135 mmol/L) versus patients remaining hyponatremic (*n* = 5) after empagliflozin treatment. Median BDNF levels at baseline were 13.14 ng/mL. After treatment, median BDNF was 14.39 ng/mL in patients reaching normonatremia (median delta BDNF +2.01 ng/mL) and 11.97 ng/mL in hyponatremic patients (median delta BDNF −2.33 ng/mL). Statistics: Wilcoxon Signed‐Rank test, *n* = 9 participants, *p* = .56, für delta BDNF *p* = .29.

The exploratory subgroup analysis assessing differences between participants reaching normonatremia (sodium >135 mmol/L), compared to participants remaining hyponatremic after the empagliflozin treatment period are shown in (Figure 5). A trend toward greater BDNF increase in the group reaching normonatremia was observed (median delta BDNF +2.01 ng/mL), which was not seen in patients remaining hyponatremic (median delta BDNF −2.33 ng/mL); however, this difference did not reach statistical significance (*p* = .29).

Baseline total MoCA score was 23.0 [21.0–24.8], with an executive function subscore of 2.5 [2.0–3.3]. In this secondary analysis, no difference in MoCA scores was observed between empagliflozin and placebo phase (empagliflozin: 26.0 [23.5–27.0], executive function subscore 4.0 [2.5–4.5], placebo: 27.0 [23.5, 28.5], executive function subscore 4.0 [2.5–4.0]). No association was observed between change in BDNF levels and change in MoCA total score or executive subscore (Figure S1, Supporting Information).

## 4 DISCUSSION

The results of our study indicate for the first time that sodium increase is associated with an increase in the neurocognitive biomarker BDNF. Given the negative effects of chronic hyponatremia on cognitive function, our findings highlight the positive impact of sodium normalization on cognitive function, with BDNF representing a promising peripheral biomarker of neuroprotection and synaptogenesis.

In chronic hyponatremia (defined as >48 h duration), the brain adapts gradually by expelling intracellular ions and organic osmolytes, a protective process of brain cells, known as regulatory volume decrease.^25^ This process helps to maintain cellular volume and preserve neuronal function and explains the rather mild symptoms of chronic hyponatremia. However, there is growing evidence that chronic hyponatremia is not asymptomatic but is associated with cognitive impairments that resemble attention deficits seen with moderate alcohol intake.^21^ Importantly, correction of hyponatremia has shown to be able to improve clinical symptoms and neurocognitive function, underlining the causal relationship between sodium levels and cognitive function.^15^ Specifically, cognitive improvements after hyponatremia correction have been shown in a prospective case–control study of 19 patients, investigating “go/no‐go” performance and another randomized‐controlled study investigating psychomotor speed with the Morse Tapping Test in hyponatremia correction with tolvaptan (vasopressin antagonist) treatment.^20^, ^22^ In line with this, our original study showed that correction of hyponatremia with empagliflozin leads to improved neurocognitive function with an increase of 1.16 (95%CI, 0.05–2.26, *p* = .04) in the total MoCA score with the strongest effect in the MoCA executive function.^23^

The fact that participants presented with only mild hyponatremia may have led to only subtle cognitive impairments and therefore limited improvements in MoCA performance. Our secondary analysis could not confirm significant changes in the MoCA test between groups, which potentially is due to the even smaller sample size of only nine patients with sufficient data (as compared to 14 patients in the original trial).

However, next to this first evidence deriving from a clinical study, in vivo studies^26^ also suggest that correction of hyponatremia improves cognitive function,^22^ brain structure, and neuronal activity—particularly in the hippocampus.^27^ The neurotrophin BDNF has been shown to be critical for memory, learning, and synaptic plasticity in the central nervous system, with peripheral levels increasing during antidepressant treatment^12^, ^13^, ^28^ and after physical activity.^14^ However, BDNF in hyponatremia correction had never been investigated. To our knowledge, our study is the first to show that BDNF levels increase following correction of hyponatremia, suggesting a causal role for BDNF in the cognitive improvements observed after hyponatremia correction. Based on our findings, it can be hypothesized that even slight improvements in sodium levels affect BDNF levels, suggesting benefits of sodium normalization on cognitive function. Interestingly, patients reaching normonatremia showed the highest increase in BDNF levels while patients remaining hyponatraemic showed no change in BDNF levels. Notably, the observed increase in BNDF following hyponatremia correction in our study (~9%) was comparable to the mean increases reported following antidepressant treatment (~12%).^29^

Remarkably, the strongest increase in BDNF levels upon sodium increase was seen under empagliflozin treatment in our study. Exploratory analysis showed that empagliflozin markedly strengthened the positive relationship between sodium levels and BDNF increase, suggesting a treatment‐specific effect of SGLT2‐inhibitors. This interaction effect remained robust after adjustment for age, sex, and baseline sodium. Recent reviews and meta‐analyses, mainly focusing on SGLT2‐inhibitor effect in patients with diabetes, have suggested neuroprotective effects of SGLT2‐inhibitors, as shown by improvements in cognitive outcomes and reduced dementia risk.^30^, ^31^ The positive effects are mainly thought to be influenced by reduced inflammation and oxidative stress. Therefore, these protective effects on cognition can also be speculated in hyponatremia correction with SGLT2 inhibitor treatment, potentially explaining the more profound increase of BDNF in sodium correction upon SGLT2 inhibitor treatment. To our knowledge, data on the impact of SGLT2‐inhibitors on serum BDNF is scarce. The only available evidence for SGLT2 inhibitor effects specifically on BDNF is limited to preclinical animal models. The available data is in line with our study, as SGLT2 inhibitors have shown to modulate BDNF gene expression and increase BDNF levels in the brains of diabetic mice, particularly in the prefrontal cortex and the hippocampus.^30^, ^32^ Although the precise mechanism remains unknown, current evidence from mouse models suggests that SGLT2 inhibitor‐induced upregulation of BDNF is probably secondary to improved cerebral insulin signaling, reduced oxidative stress,^32^ and lower neuroinflammation, and possibly enhanced through glucagon‐like peptide‐1 (GLP‐1) and hypoxia inducible factor 1α (HIF‐1α) signaling, all of which converge on BDNF gene expression.^33^, ^34^

Given the predominance of female participants in our cohort, the potential influence of sex‐specific or X‐linked genetic factors on the response to SGLT2 inhibitors warrants consideration. Although first data suggests that empagliflozin might exert epigenetic effects through modulation of microRNAs,^35^ larger data are needed to assess the role of X‐linked loci in determining responsiveness to SGLT2 inhibitors. In our study, we observed no sex‐related differences in treatment response, although our study was not powered to detect subtle sex‐specific effects.

Taken these findings together, our pilot study suggests additional benefits of SGLT2‐inhibitors on the cognitive marker BDNF in hyponatremia correction, warranting further investigation in larger cohorts.

Some limitations of our study should be addressed. As the main study was not powered for this secondary analysis, the results are based on a small sample size and especially the subgroup analyses (such as normonatremia vs. persisting hyponatremia, sex, age) are underpowered and should be seen as exploratory and hypothesis‐generating. However, the crossover design of our study enabled stable comorbidities and medication regimes across both groups, allowing for interpretation of individual changes.

The key strength of this study is the prospective, randomized‐controlled cross‐over design with standardized sample collection, conducted in the morning under fasting conditions, which helps control for diurnal and metabolic variability in BDNF levels.

In summary, our findings suggest that correction of hyponatremia is associated with an increase in the neurocognitive biomarker BDNF. This underscores the impact of sodium normalization on cognitive health, with BDNF representing a promising peripheral biomarker of neurocognitive protection and potential synaptogenesis. Future larger studies are needed to further clarify the role of serum BDNF in the clinical management of hyponatremia and across different treatment approaches.

## AUTHOR CONTRIBUTIONS

**Julia Beck:** Project administration; writing – review and editing; methodology; formal analysis; visualization. **Julie Refardt:** Conceptualization; project administration; writing – review and editing; supervision. **Eszter Kustos‐Tóth:** Formal analysis; writing – original draft; methodology; visualization. **Mirjam Christ‐Crain:** Conceptualization; funding acquisition; writing – review and editing; project administration; supervision. **Lucia Seeger:** Formal analysis. **Sophie Monnerat:** Writing – review and editing; project administration; data curation. **Cemile Bathelt:** Data curation; writing – review and editing; project administration.

## FUNDING INFORMATION

This study was supported by the Swiss Endocrine Society (Young Investigator grant to J. Refardt); by the University Hospital Basel and by the Swiss National Science Foundation (SNF‐162608 to M. Christ‐Crain; SNF‐199391 MD‐PhD fellowship to S. Monnerat). The funders had no role in the design and conduct of the study; collection, management, analysis, and interpretation of the data; preparation, review, and approval of the manuscript; and decision to submit the manuscript for publication.

## CONFLICT OF INTEREST STATEMENT

The authors declare no conflicts of interest.

## Supporting information


**Table S1.** Statistics linear mixed‐effects model to assess the relationship between sodium change and BDNF change, *n* = 18 observations.
**Figure S1.** Association between change in MoCA scores and BDNF change. No significant association was found between change in MoCA total score/MoCA executive function subscore and BDNF change.

## Data Availability

We may share de‐identified, individual participant‐level data that underlie the results reported in this article and related documents, including the study protocol and the statistical analysis plan. Data will be available with the publication of our main manuscript on receipt of a request detailing the study hypothesis and statistical analysis plan. All requests should be sent to the corresponding author. The steering committee of this study will discuss all requests and decide based on the scientific rigor of the proposal whether data sharing is appropriate. All applicants are asked to sign a data access agreement.
