# Supplementary material for: Hyponatremia correction is associated with increased brain‐derived neurotrophic factor levels: A pilot secondary analysis of a randomized, double‐blind, placebo‐controlled, crossover trial
Source: J Neuroendocrinol. 2026 Jul 28;38(8):e70238. doi: 10.1111/jne.70238 (PMC13416232; doi:10.1111/jne.70238)
Supplement: Supplementary file 1 — Table S1. Statistics linear mixed‐effects model to assess the relationship between sodium change and BDNF change, n = 18 observations. Figure S1. Association between change in MoCA scores and BDNF change. No significant association was found between change in MoCA total score/MoCA executive function subscore and BDNF change. [file JNE-38-e70238-s001.docx]

SUPPLEMENTARY

**Hyponatremia correction is associated with increased brain-derived neurotrophic factor levels (BDNF)**

**–**

**Secondary analysis of a randomized, double-blind, placebo-controlled, crossover trial**

Eszter Kustos-Tóth *^12^, Julia Beck *^12^, Lucia Seeger ^12^, Sophie Monnerat ^12^, Cemile Bathelt ^12^, Julie Refardt *^12^, Mirjam Christ-Crain *^12^

^1^ Dept of Endocrinology, Dept of Internal Medicine, University Hospital Basel, Switzerland

^2^ Dept of Clinical Research, University Hospital Basel, University of Basel, Switzerland

* These authors have contributed equally and share first authorship

* These authors have contributed equally and share senior authorship

**Corresponding author:**

Mirjam Christ-Crain

University Hospital Basel

Dept. of Endocrinology, Diabetology und Metabolism

Petersgraben 4, 4031 Basel, Switzerland

Mirjam.christ-crain@usb.ch

| **Fixed Effects** | **Estimate** | **Std. Error** | **df** | **t value** | **Pr(>\|t\|)** |
| --- | --- | --- | --- | --- | --- |
| **(Intercept)** | -47.19107 | 57.55538 | 11 | -0.820 | 0.4297 |
| **Sodium Delta** | 0.70727 | 0.31718 | 11 | 2.230 | 0.0475 * |
| **Arm: Empagliflozin** | -4.48677 | 2.33319 | 11 | -1.923 | 0.0807 |
| **Sex- Female** | 2.64185 | 2.33134 | 11 | 1.133 | 0.2812 |
| **Age** | -0.07809 | 0.09640 | 11 | -0.810 | 0.4351 |
| **Baseline Sodium** | 0.41481 | 0.47588 | 11 | 0.872 | 0.4020 |
| **No antidepressive Therapy** | -1.20056 | 2.63757 | 11 | -0.455 | 0.6578 |

***Suppl. Table 1:* Statistics linear mixed-effects model to assess the relationship between sodium change and BDNF change, n=18 observations**


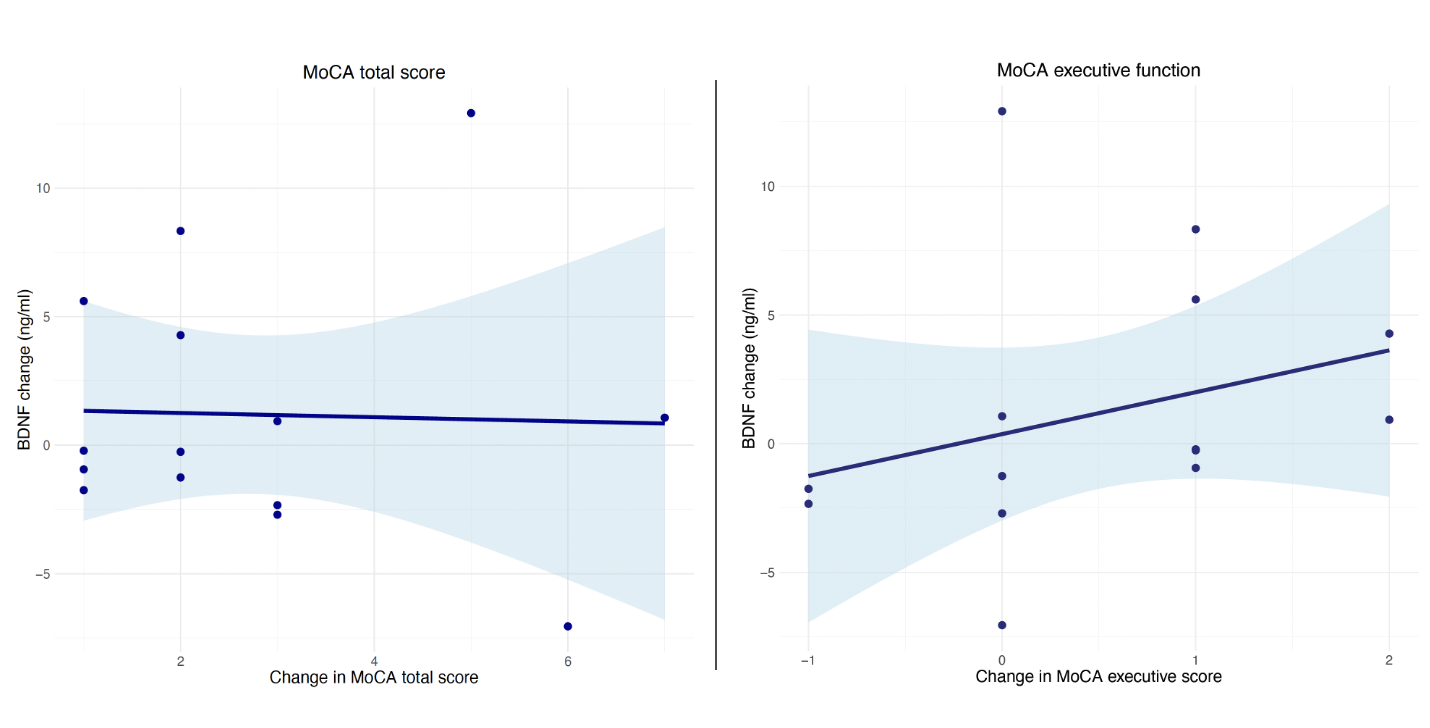


***Suppl. Figure 1: Association between change in MoCA scores and BDNF change***

*No significant association was found between change in MoCA total score/ MoCA executive function subscore and BDNF change.*
